# Boric Acid as A Low-Temperature Graphitization Aid and Its Impact on Structure and Properties of Cellulose-Based Carbon Fibers

**DOI:** 10.3390/polym15214310

**Published:** 2023-11-02

**Authors:** Tobias Hückstaedt, Jens Erdmann, André Lehmann, Robert Protz, Johannes Ganster

**Affiliations:** Material Development and Structure Characterization, Biopolymers, Fraunhofer Institute for Applied Polymer Research IAP, Geiselbergstraße 69, 14476 Potsdam, Germany; jens.erdmann@iap.fraunhofer.de (J.E.); andre.lehmann@iap.fraunhofer.de (A.L.); robert.protz@iap.fraunhofer.de (R.P.); johannes.ganster@iap.fraunhofer.de (J.G.)

**Keywords:** carbon fibers, cellulose, viscose, renewable resources, boric acid, boron, catalytic effect, graphitization

## Abstract

In the present paper, a scalable, economically feasible, and continuous process for making cellulose-based carbon fibers (CFs) is described encompassing precursor spinning, precursor additivation, thermal stabilization, and carbonization. By the use of boric acid (BA) as an additive, the main drawback of cellulose-based CFs, i.e., the low carbon yield, is overcome while maintaining a high level of mechanical properties. This is demonstrated by a systematic comparison between CFs obtained from a BA-doped and an un-doped cellulose precursor within a temperature range for carbonization between 1000 and 2000 °C. The changes in chemical composition (via elemental analysis) and physical structure (via X-ray scattering) as well as the mechanical and electrical properties of the resulting CFs were investigated. It turned out that, in contrast to current opinion, the catalytic effect of boron in the formation of graphite-like structures sets in already at 1000 °C. It becomes more and more effective with increasing temperature. The catalytic effect of boron significantly affects crystallite sizes (*L_a_*, *L_c_*), lattice plane spacings (*d*_002_), and orientation of the crystallites. Using BA, the carbon yield increased by 71%, Young’s modulus by 27%, and conductivity by 168%, reaching 135,000 S/m. At the same time, a moderate decrease in tensile strength by 25% and an increase in density of 14% are observed.

## 1. Introduction

Carbon fibers (CFs) combine high strength and stiffness with a low density in a unique way. This makes them highly attractive as a reinforcing material for light-weight constructions, especially in the mobility sector for reducing fuel consumption by weight savings. They are also used in other end-use applications, including aerospace, wind turbine blades, hydrogen pressure tanks, and sports and medical equipment [1,2]. Almost all CFs used in these applications are made from either polyacrylonitrile copolymers (PAN) or mesophase pitch which together account for a market share of between 96 and 98% [3]. However, CFs based on natural resources such as cellulose have recently attracted much attention due to their high potential for having a significantly lower carbon footprint and lower possible costs [3,4,5].

At the start of their development, CFs were made from renewable polymers, namely cellulose. In 1964, Union Carbide introduced “Thornel 25”, the first commercially available, cellulose-based CF with a Young’s modulus of approximately 170 GPa. Later on, “Thornel 50”, “Thornel 75”, and “Thornel 100” were produced, having Young’s moduli ranging from 345 to 690 GPa and tensile strengths from 1.97 to 3.95 GPa [1]. Thus, the technological capability for the production and the marketability of cellulose-based CFs with outstanding properties were proven. However, in competition with the emerging PAN-based fibers, high prices caused by low carbon yield and the need for hot stretching at temperatures of 2500 °C or higher (stretch-graphitization) led to the termination of production in 1978. The ex-PAN fibers had established themselves in the market as an alternative with good mechanical properties and significant economic advantages and rapidly became the absolute dominating precursor system [1,6]. Nowadays, PAN-based precursors are assessed more critically for their non-renewable origin and the toxicity of monomers, e.g., acrylonitrile and gases, e.g., hydrocyanic emitted during the production process. Moreover, the progressive exothermic cyclization reactions occurring in stabilization carry the risk of accidents during production [1]. Therefore, cellulose-based precursors, as an alternative, came into focus again [4]. Nevertheless, the drawbacks of cellulose-based CFs like poor yield and the need for stretch-graphitization still need to be overcome. The most relevant approaches to both problems are described below.

Cellulose can be formed into continuous filaments using solution spinning methods like the viscose and Lyocell process [7,8]. Since these yarns can be converted without melting, they are potential candidates for CF precursors [1,9,10]. This potential is limited by the chemical composition of cellulose (C_6_H_10_O_5_), which corresponds to a carbon content of only 44.4 wt%. As the final CFs consist almost exclusively of carbon, hydrogen and oxygen must be removed during the production, taking care that no further carbon atoms are stripped away by hydrogen and especially oxygen in the form of, e.g., carbon monoxide, carbon dioxide, aldehydes, organic acids or tars, which would reduce the yield even further [9,11,12]. In order to achieve the maximum possible yield of 44.4 wt%, extremely slow heating rates [9,13], reactive atmospheres [1,13], and reduced pressure during conversion [14] were investigated. Moreover, suitable additives like Lewis acids and flame-retardants, e.g., ammonium tosylate, ammonium dihydrogen phosphate [3,9,15], diammonium hydrogen phosphate, phosphoric acid, ammonium sulfate, boric acid (BA) [9,13,16,17], or carbon-rich fillers, such as carbon black [12] and lignin [18,19,20,21], were tested. The latter cellulose-lignin precursors show outstanding potential due to their excellent spinnability at high lignin levels [4,22,23] and a high mass yield after thermal conversion of up to 44 wt% [4,24,25,26,27,28]. 

For cellulose-based precursors without lignin, the mass yield at 1400 °C can be increased from 17 to 37 wt% by using ammonium tosylate [3]. Investigating additives for cellulose to improve flame retardancy, Hirata and Werner found similar effects with boric acid by thermogravimetric analysis (TGA) using purified cotton linters [29]. The yield of cellulose linters heated up to 500 °C in a nitrogen atmosphere increased from 9 to 27 wt% when 3.4 wt% boric acid was used. However, neither the TGA-conditions (heating rates or atmosphere) nor the tested cellulose (linters) are comparable to the present case. To the best of the authors’ knowledge, there are no publications dealing with the mass yield during the production of cellulose-based CFs even though boron-containing compounds have long been known as an effective flame retardant [30] for cellulose.

As to the second issue, cellulose-based CFs with mechanical properties comparable with ex-PAN fibers could only be produced at temperatures above 2500 °C in combination with stretching [1]. Without these measures, the tensile strengths of cellulose-based CFs typically range between 0.5 and 1.5 GPa [5,31]. This is true for systems without further additives. However, when ammonium tosylate is used as an additive, cellulose-based CFs can be produced with tensile strengths of up to 2.0 GPa at temperatures as low as 1400 °C [3] or even with 2.8 GPa when processed with reduced pressure during stabilization [14]. In addition to tensile strength, the Young’s modulus is crucial for the use of CFs in lightweight applications. For non-graphitized cellulose-based CFs, the Young’s modulus is typically much less than 100 GPa. However, by applying a draw ratio of 1.12 during carbonization at 1400 °C after stabilization with reduced pressure, [14] obtained a modulus of 112 GPa. Still, this is far from the PAN-based standard modulus CFs, such as the T300 grade from Toray, having 230 GPa [1].

In general, catalytic effects can provide a clue for reducing the carbonization temperatures to a PAN-like level. Such effects are known for various metals such as Ni, Co, Fe, Pt, Mo, and Cr [32,33] and, in particular, for boron-containing compounds [34,35,36]. The unique nature of boron as a catalyst is due to its solubility and mobility in carbonaceous structures [36,37,38]. Although the details of the mechanism are discussed controversially, there is a consensus that boron catalysis graphitization [34,35,36,37,38]. Barton et al. showed that the use of 0.5 M boric acid doubled the Young’s modulus to 201 GPa and tensile strength to 2.4 GPa of polyethylene-based CFs processed at 1800 °C, with respect to an un-doped reference [35]. Structurally, a catalytic effect was also demonstrated for cellulose-based CFs exposed to boron vapor with X-ray diffraction, Raman spectroscopy, scanning electron microscopy, solid state NMR, high-resolution transmission electron microscopy, and TGA by [34]. Unfortunately, no mechanical properties were reported.

From the above it seems worthwhile to investigate in more detail the catalytic effect of boron or a boron-containing substance, such as boron acid, with the aim of increasing carbon yield and improving the Young’s modulus of cellulose-based CFs.

## 2. Materials and Methods

### 2.1. Raw Materials

A softwood pre-hydrolysis kraft dissolving wood pulp (Georgia-Pacific, Atlanta, GA, USA), with a degree of polymerization of DPCuoxam = 611 and an α-cellulose content of 96.5% (own measurements) was used throughout. Xanthation was performed with carbon disulfide with 99.9% purity (Merck, Rahway, NJ, USA). The additive solution was produced from demineralized water and orthoboric acid with a purity of 99.8% (VWR Chemicals, Radnor, PA, USA).

### 2.2. Preparation of the Viscose Spinning Solution

A cellulose content of 9.1 wt% and an alkali content of 6.7 wt% was employed for the spinning solution. The pulp was cut into sheets (18 × 22 cm) which were placed into the steeping chamber of a Blaschke plant. Alkali lye having a concentration of 18 wt% was slowly introduced from bottom to top into the alkalization chamber. Subsequently, steeping was performed for 50 min at 35 °C. The resulting sheets of alkali cellulose (AC) were then pressed out with a factor of 2.9. The resulting AC, containing 36.9 wt% cellulose and 14.5 wt% alkali, was then destructured in a Blaschke kneader system. The resulting AC was aged at 35 °C to reach a DP Cuoxam of 340. Xanthation was carried out in a so-called baratte. To this end, the AC was put into the baratte and a vacuum was applied. Carbon disulfide (32 wt% related to the cellulose) was flushed into the de-pressurized reaction chamber for the xanthation reaction, which took 90 min at 28 °C. During the reaction, the pressure decreased to the starting value, indicating that the complete carbon disulfide was used up for the xanthation reaction.

The resulting yellowish sodium cellulose xanthate had a γ-value of 51.4 and was dissolved in sodium hydroxide solution after pre-cooling the solution to 6 °C; the sodium cellulose xanthate was introduced into the dissolving equipment (rotor/stator dissolving geometry of 5 l volume). The dissolution process was finished after 120 min and the temperature was kept at 6–8 °C. The spinning dope was filtered through a 20 µm metal fleece filter. By adjusting time and temperature, the maturity was set to a degree Hottenroth of 9.0 °H.

### 2.3. Spinning of Multifilament Yarn

During the wet-spinning process, the viscose spinning solutions were kept at 20 °C in a reservoir. From there, the viscose was transported to the spinning nozzle using a spinning pump with a volume throughput of 1.2 cm^3^ per rotation. A spinneret with 1000 holes was used, with each hole having a diameter of 60 µm. The aqueous spinning bath contained 90 g/L sulfuric acid, 240 g/L sodium sulfate, and 23 g/L zinc sulfate and was kept at 40 °C. The precipitated multifilament yarn was drawn from the nozzle by a take-up roller. Subsequently, the yarn passed a decomposition bath containing 20 g/L sulfuric acid in water at a temperature of >98 °C, followed by washing the yarn on Nelson-type rollers using demineralized water at 60 °C. Finally, the washed yarn was dried under isometric conditions using drying rollers at 90 °C. Before the drying step, one yarn sample was impregnated inline with aqueous boric acid solution (4 wt%) in a treatment bath. Jet-stretch and draw factor were adjusted to 0.9 and 1.2, respectively, at a spinning speed of 15 m/min. In such a way two bobbins were manufactured each containing several hundred meters of wound yarn, one as a reference and one impregnated with boric acid solution.

### 2.4. Stabilization and Carbonization

Both the unmodified (reference) and the boric acid impregnated (BA-doped) endless cellulose yarn were continuously transported through the following three different tube furnaces:

- Stabilization furnace;
- Low-temperature (LT) furnace;
- High-temperature (HT) furnace.

Stabilization and LT carbonization were carried out in a combined process using a line speed of about 0.03 m/min, resulting in a retention time of approximately 1 h per furnace (Figure S1). The stabilization process was performed at about 270 °C under air flow (6 L/min) and the LT carbonization at a maximum temperature of 1000 °C for 15 min under nitrogen atmosphere (5 L/min). During the LT carbonization process, the fiber tension was set to 1.25 cN/tex for both the reference and the BA-doped counterpart. The resulting intermediate CFs were wound on bobbins. The final HT carbonization process was carried out under an argon atmosphere by applying 8 different temperature profiles shown in Figure S2. The intermediate CF yarns were continuously transported through the HT furnace without applying any fiber tension, such that fiber shrinkage was allowed (Figure S3). In this way, the samples were annealed to a maximum temperature between 1000 and 2000 °C. The fiber speed was set to 0.165 m/min, which corresponds to a retention time at Tmax (zone 4 and 5) of approx. 7 min. After passing through the heating zones, the CFs were cooled in the jacket-cooled and argon-purged lock.

### 2.5. Characterization

#### 2.5.1. Linear Density

Linear density for both precursors and CFs was determined gravimetrically in accordance with standard DIN EN ISO 2060:1995 and DIN EN ISO 1889:2009-10, respectively. Therefore, the mass of a conditioned strand m_c_ in g with a length of *L* = 1 m was measured with the XS05 Dual Range balance from Mettler Toledo (Columbus, OH, USA). The linear density *Tt* in tex was calculated as follows:

(1) $$Tt=\frac{m_{c}\cdot \;10^{3}}{L}$$

#### 2.5.2. Thermogravemetry

The thermogravimetric analyzer TGA2 LF/1100/885 from Mettler Toledo was used to measure the mass loss as a function of the temperature. Approx. 6 mg of chopped fibers were placed in an alumina crucible and heated from room temperature up to 1100 °C at a rate of 10 K/min in an N_2_-atmosphere (20 mL/min).

#### 2.5.3. Mass Loss and Carbon Yield

The mass loss was calculated from the weight of the heat treated yarn sample related to the weight of the untreated precursor yarn taking the shrinkage or drawing into account. The carbon yield was determined as the percentage of carbon contained in the converted fibers relative to the amount of carbon in the initial precursor, both determined by elemental analysis (see below).

#### 2.5.4. Fiber Cross Section

Images of the fiber cross sections were recorded using the scanning electron microscope GeminiSEM 300 from Zeiss (Oberkochen, Germany) at an accelerating voltage of 5 kV. Before SEM imaging, the fiber samples were cut, fixed to a sample carrier using conductive carbon tape, and finally sputtered with a 4 nm platinum layer. Fiber cross-sectional areas were measured manually with the software ImageJ (1.54d 30) for at least 20 different fiber cross sections taken from the SEM images at a magnification of at least 2000×. The average cross-sectional area A and the corresponding errors were used in the calculation of the Young’s modulus and the tensile strength, as well as the electrical resistivity.

#### 2.5.5. Density 

The density of the fibers was determined at room temperature in accordance to ISO 10119:2020 method C using a density-gradient column (DGC) filled with the liquids acetone and dibromomethane.

#### 2.5.6. Elemental Analysis (EA)

The elemental composition (C, H, N, S) was measured with the FlashEA 1112 from Thermo Scientific (Waltham, MA, USA) on chopped fibers. The boron (B) concentration was determined via ICP-OES using an ICP OES Optima 2100 DV from Perkin-Elmer (Waltham, MA, USA) after nitric acid digestion.

#### 2.5.7. Structural Properties (WAXS)

The crystalline phase of the fibers was analyzed with the wide angle X-ray scattering (WAXS) system D8 Advance from Bruker (Billerica, MA, USA) equipped with a Cu-Tube (*λ* = 0.15419 nm) and a Si-strip detector (LYNXEYE XE-T). For the calibration, the standard NIST SRM 1976 in the Bragg–Brentano geometry was used.

#### 2.5.8. Crystallite Dimensions

The crystallite size *L_a_* (persistence length parallel to the fiber axis) and *L_c_* (graphite bundle thickness), as well as the lattice plane spacing *d*_002_, were determined with isotropic samples in transmission geometry. Thus, approximately 10 mg of the chopped CFs were milled with a vibrating ball mill and filled in capillaries having a diameter of 1 mm. A *θ*-2*θ* scan was performed from 2*θ* = 5 to 90° using a focusing Goebel mirror and the silicon detector in the 1D mode. All diffractograms were corrected for background scattering, absorption, and polarization [39,40,41,42]. For the determination of *L_c_* and *d*_002_, the (002) reflection was analyzed using a split Pseudo-Voigt profile [43], whereas for *L_a_*, the (11) reflection, a split Pseudo-Voigt profile combined with a broad Gaussian background profile was applied. Based on the full width at half maximum *β* and the Bragg angles *θ* of the (002) and the (11) reflections, the crystallite sizes were calculated using the Sherrer equation (Equation (2)) [44]. The lattice plane spacing was calculated using Bragg’s law (Equation (3)). According to [45,46] the constants *K* = 0.89 for *L*_c_ and *K* = 1.84 for *L_a_* were used.

(2) $$L=\frac{K\times \lambda }{\beta \;\cos\theta }$$

(3) $$d_{002}=\frac{\lambda }{2\;\sin\theta }$$

#### 2.5.9. Crystalline Orientation

The degree of preferred relative orientation (*OG*) and the integrated orientation parameter (*OGI*) were determined from azimuthal scans of the (020) and the (002) reflections of the precursors and the CFs, respectively. The fibers were prepared unidirectionally on a sample holder and analyzed in transmission geometry using the WAXS system equipped with a polycap combined with a collimator (2 mm opening) and the 0D mode of the detector. After correcting the intensities for background scattering, absorption, and polarization [39,40,41,42], the *OG* and *OGI* were calculated via Equations (4) and (5), respectively, including the half width at full maximum (*FWHM*) and the sum of the areas of both peaks (*I_ori_*) in relation to the total area (*I_total_*):

(4) $$OG=\frac{180{}^{\circ}-FWHM}{180{}^{\circ}}$$

(5) $$OGI=\frac{I_{ori}}{I_{total}}$$

For the determination of the *FWHM*, *I_ori_* and *I_total_*, the corrected azimuthal scans, were fitted in the range from *φ* = 0 to 360° by applying two independent Gaussian functions on a constant background.

#### 2.5.10. Electrical Conductivity 

For determining the electrical conductivity, the volume resistivity *S_f_* (its reciprocal value) was measured for a strand of 1000 fibers of 30 cm in length. The strand was contacted with metal clamps at four equidistant positions, with a distance *L_f_* of 5 cm. In this way, the resistance between two neighboring contacts *R_f_* was determined with a D. C. Milli-Ohmeter GOM-805 from GW Instek by applying a current of 10 mA. The measurements were conducted in accordance with BS ISO 13931, taking into account the number of filaments n and fiber cross-sectional area A, as shown in Equation (6).

(6) $$S_{f}=\frac{n\cdot A\cdot R_{f}}{L_{f}}\cdot 10^{3}$$

#### 2.5.11. Mechanical Properties

The tensile testing of single filaments was performed on a ZwickRoell (Ulm, Germany) Z020 Retro Line system based on ASTM D 3822 in standard atmosphere (23 °C and 50% RH) according to DIN EN ISO 291. After conditioning the samples for 24 h in the testing lab, at least 15 single filaments were separated from the yarn and tested. The following testing parameters were used: preforce of 0.1 cN, clamp distance of 20 mm, and testing speed of 10 mm/min. The recorded force-strain curves were related to the average cross-sectional area A (from SEM) to obtain values for Young’s modulus in GPa, tensile strength in MPa, and the elongation at break in percent.

## 3. Results and Discussion

As described in the section “Materials and Methods”, two cellulose-based precursors were produced, which differ only in an aqueous boric acid impregnation. The corresponding properties are listed in Table 1.

Both precursors were converted to CFs in the same way by performing three successive thermal treatment steps: stabilization up to 270 °C, low-temperature carbonization (LT) up to 1000 °C, and finally high-temperature carbonization (HT) up to 2000 °C. While the processing for stabilization and LT carbonization remained unchanged, the maximum temperature in the HT carbonization was varied in eight steps between 1000 and 2000 °C (Figure S3).

In the first part of this section, mass balance effects, as well as physio-chemical structural changes of the carbon fibers, will be presented. Macroscopic properties, such as fiber cross section, density, and electrical and mechanical performance, will be discussed in the second part. In doing so, the interlinkage of process parameters, structural changes, and resulting properties of the CFs will be discussed. 

### 3.1. Mass Balance Effect

**Figure 1:**
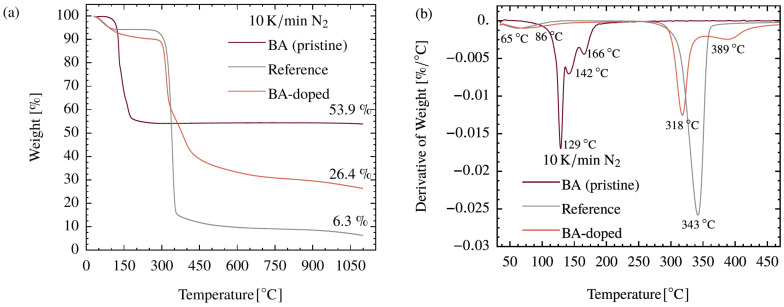
TGA (**a**) and DTG (**b**) curves for cellulose (Reference), BA-doped precursor and pristine boric acid (BA).

TGA measurements were performed to evaluate the efficiency of the BA doping and the activity regarding the BA–cellulose interaction. The reference precursor and the BA-doped counterpart, as well as pristine boric acid, were analyzed (Figure 1). BA itself shows a main mass loss in the temperature range between 100 and 220 °C to a residual mass of 53.9 wt% and then remains constant up to 1100 °C. The corresponding DTG curve clearly shows three different steps which can be assigned to the reaction of BA (B(OH)_3_) to boron trioxide (B_2_O_3_). The dehydration takes place via the intermediate products metaboric acid (HBO_2_) formed at approximately 129 °C and tetraboric acid (H_2_B_4_O_7_) formed at approximately 142 °C and is completed at 220 °C [47]. 

For the BA-doped precursor, these dehydration steps cannot be observed. Instead, main mass losses at 65 °C, 318 °C, and 389 °C are found, resulting in a residual mass of 26.4 wt% at 1100 °C. The BA-doped system differs in two major aspects from the reference: On the one hand, the main mass loss at approximately 340 °C is shifted by about 25 K to lower temperatures and is followed by an additional peak at 389 °C. On the other hand, the residual mass at 1100 °C is nearly four times higher. These findings indicate a strong interaction activity between the incorporated BA and cellulose. Such effects are also known from the literature [1,13,27,29,48] and were explained by the catalytic dehydration of cellulose in the presence of BA. This dehydration, taking place at approximately 318 °C, removes “chemically bound water” (cellulose is built up of C_6_(H_2_O)_5_ units) that is then no longer available for generating carbon containing, volatile byproducts (via, e.g., the undesirable levoglucosan) and the residual mass is increased [1,13,27,48].

**Figure 2:**
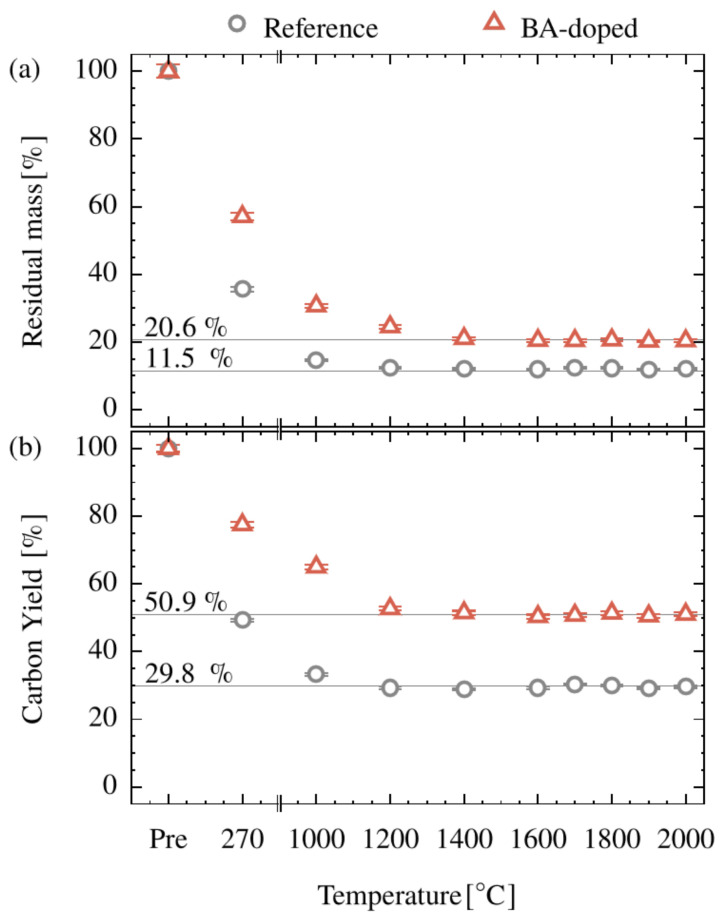
Residual mass (**a**) and carbon yield (**b**) of the cellulose and BA-doped precursor (Pre) as a function of the applied temperature during conversion.

The TGA experiments clearly show the potential of BA to reduce mass loss. This potential must now be verified for the thermal and atmospheric conditions in real CF conversion trials to assess the potential of the cellulose-based CF precursor system. Results are shown in Figure 2. As expected, the main mass decrease occurs during stabilization (270 °C) and LT carbonization. During the HT carbonization (1000–2000 °C), the mass is only slightly lowered. For a temperature higher than 1400 °C, the yield remains constant at a level of 11.5 wt% and 20.6 wt% for the reference and the BA-doped system, respectively. This indicates that the chemical conversion is finished at this point, or at least no volatile byproducts are generated anymore. The results from the final CF production and the TGA measurements show the same trend but clearly differ in numbers. For TGA the BA-induced residual mass improvement at 1000 °C is 300% (factor of four) giving 26%, whereas for CF production only a factor of two was found ending up with 20.6% residual mass. The reason for this gap can be explained by the different temperature–time regimes as well as the applied atmospheres during the conversion. However, if BA is used, the residual mass after each conversion step is significantly increased. In the above considerations, boron contributions have not been subtracted for reasons of simplicity. This shortcoming is eliminated by considering the carbon yield in Figure 2b, fully corroborating the results discussed on the basis of mass loss. It should be pointed out that these findings illustrate the importance of BA doping prior to the stabilization for the economic viability of the whole conversion.

### 3.2. Boron Concentration during Conversion

**Figure 3:**
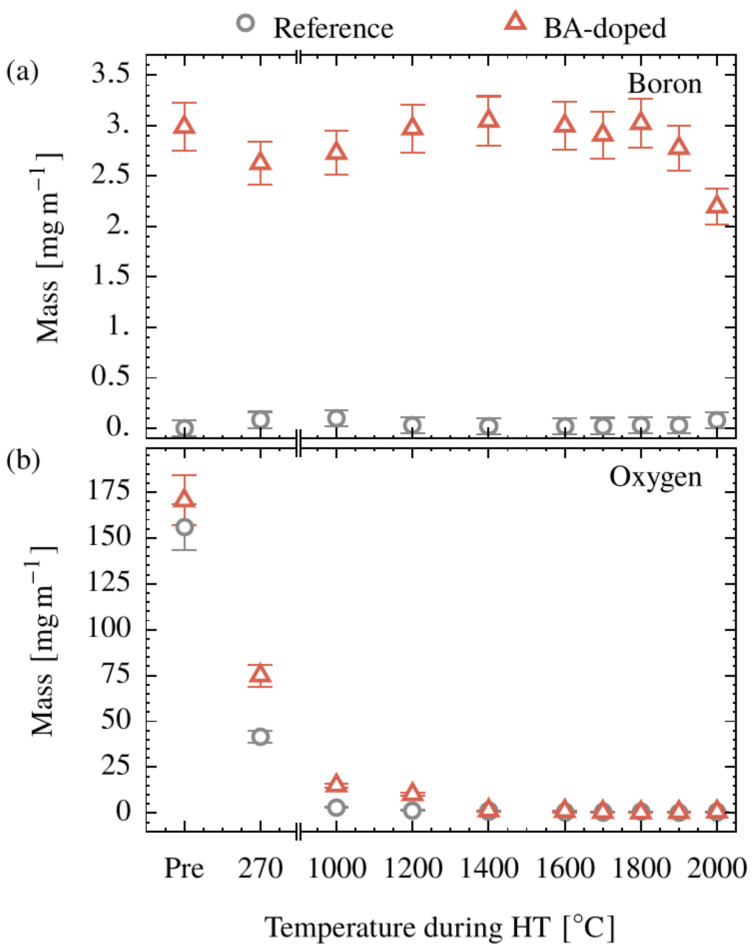
Mass per meter of precursor of boron (**a**) and oxygen (**b**) of a cellulose (Reference) and BA-doped precursor (Pre) as a function of conversion temperature.

As shown in the above, there is an unambiguous action of boron or boron-containing substances during the conversion process. The fundamental question arises if (and how long) boron is kept in the fiber during the process and if this is the case, how much? This question can be answered using ICP-OES. The result is presented in Figure 3a. It should be mentioned that the concentrations given in the figure are normalized to the length unit of the precursor (meter of precursor) since stretching (or shrinking) occurs in the process and has been monitored by the speed differences of the respective rollers. The absolute mass of boron remains constant at approximately 3 mg/m up to a temperature of 1900 °C, indicating that boron is completely preserved during the conversion process. This is changed for *T* = 2000 °C, as the absolute mass of boron decreases, which is consistent with the results of Lee and Hatori [36].

Interestingly, for the BA-doped system, significantly more oxygen was found up to 1200 °C (Figure 3b) by elemental analysis. This can be explained by the formation of boron trioxide (B_2_O_3_) and thus keeping boron in the system, a process completed at approximately 220 °C for pristine BA as shown in Section 3.1. Above a temperature of 1400 °C, the oxygen content of the BA-doped system is too low to meet the stoichiometric conditions of boron trioxide (1 wt% boron to 2.2 wt% oxygen). So, boron, at least partially, must have been transformed into another form, e.g., boron carbide, which was found via WAXS experiments.

### 3.3. Structural Properties (WAXS)

**Figure 4:**
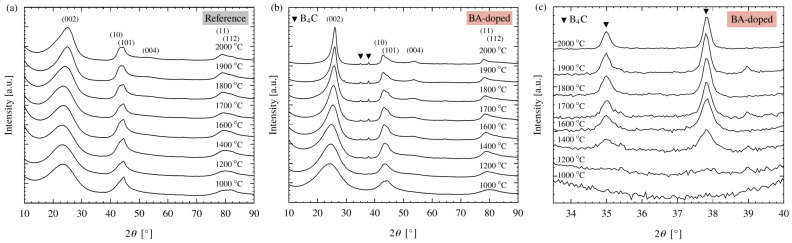
Corrected WAXS diffractograms of the CFs made from the cellulose (Reference) (**a**) and BA-doped precursor (**b**) and zoom in of the latter between 34° and 40° 2*θ* (**c**).

The corrected diffractograms of the CFs based on the cellulose and the BA-doped precursors are shown between 10 and 90° in 2*θ* in Figure 4a and Figure 4b, respectively. For both systems, a graphite-like phase is observed, including the typical (002), (10) and (11) reflections [45,49]. In general, with increasing temperature, progressive graphitization is indicated by a shift in the (002) reflection to larger angles accompanied by a reduction in half width as well as an emerging (004) reflection at about 53.8°. Obviously, this temperature-induced graphitization is much more pronounced for the BA-doped system. 

In addition to the reflections assigned to the graphite-like phase, two further small peaks at 35.0° and 37.8° are found for the BA-doped system (marked with triangles in Figure 4b). These sharp reflections, shown in detail in Figure 4c, can be assigned to an additional crystalline phase, which is most likely boron carbide (B_4_C). This assignment is in accordance with the peak positions published by Li et al. and Kakiage et al. [50,51,52] as well as with the chemical composition of the CFs. The B_4_C phase formation starts between 1200 and 1400 °C and develops up to 1600 °C. At 2000 °C, the intensity of the B_4_C phase is slightly reduced, most probably due to the sublimation of boron starting above 1900 °C, which was shown via elemental analysis.

**Figure 5:**
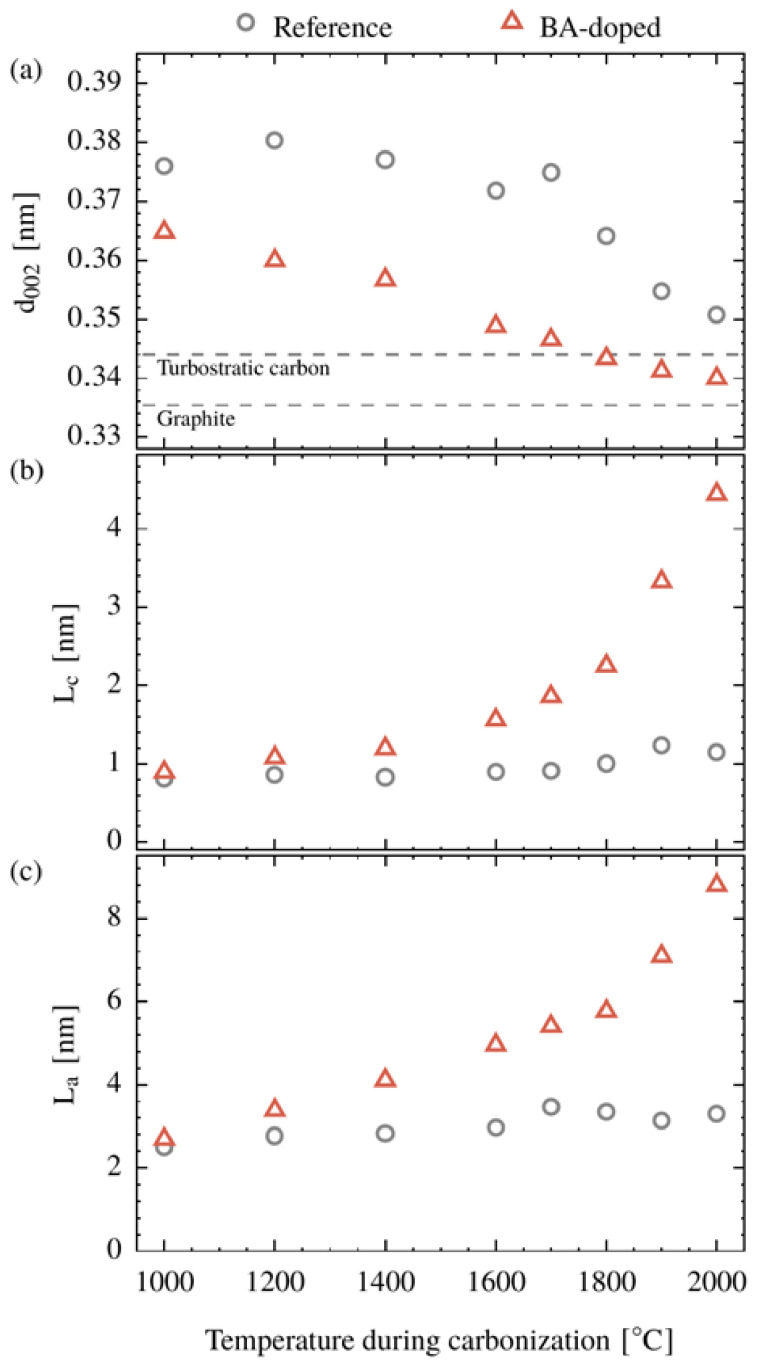
Lattice plane spacing *d*_002_ (**a**), crystallite sizes *L_c_* (**b**) and *L_a_* (**c**) of the CFs made from the cellulose (Reference) and the BA-doped precursor as a function of the carbonization temperature.

In addition to the qualitative phase analysis, the progress in graphitization as a function of temperature is quantified by the determination of the lattice plane spacing *d*_002_ and the crystallite sizes *L_c_* and *L_a_*, shown in Figure 5. In terms of *d*_002_, the two systems differ significantly over the whole temperature range. This fact implies that the effect of boron on the crystalline structure is already present at 1000 °C. With increasing temperature, the lattice plane spacing *d*_002_ of the BA-doped system decreases almost linearly between 1000 and 2000 °C, whereas for the reference system, *d*_002_ remains constant up to 1700 °C and decreases between 1700 and 2000 °C finally down to *d*_002_ = 0.351 nm. The BA-doped system has reached the region of turbostratic graphite [45] at 1800 °C and at 2000 °C, a lattice plane spacing of *d*_002_ = 0.341 nm is observed, which is remarkably low compared to the reference system.

The crystallite sizes *L_c_* and *L_a_* of the reference system (Figure 5b,c) increase slightly over the whole temperature range and reach their maximum with 1.2 and 3.3 nm, respectively, at 2000 °C. In contrast, when boron is present, crystallite growth is strongly increased, in particular for temperatures higher than 1800 °C. At 2000 °C, the crystallite dimensions reach a maximum of *L_a_* = 8.8 nm and *L_c_* = 4.4 nm, which correspond to a volume 35 times larger than that of the reference. 

Considering the significant impact of boron on the structure formation and despite the low concentration (1 wt% in the precursor), a strong catalytic effect was demonstrated. There is a general consensus that boron and some boron-containing compounds have such an effect on the graphitization of carbon materials [34,35,36,37,38]. However, the start temperatures found in the literature are above 1900 °C throughout (with one exception, see below). So, Hagio et al. found a catalytic effect at 2000 °C for BA-soaked graphite (0.6 wt% B) [53], Murty et al. at 1900 °C on various cokes dry blended with boron powder in concentrations of 0.5 up to 5.0 wt% [54], and Lee and Hatori at about 2300 °C on boron-doped cellulose-derived carbons based on a mixture of cellulose and boron [36]. In all of these cases, the catalytic effect can only take place after the sublimation of boron, which typically occurs at temperatures above 1800 °C [55]. The exception is a sulfonated polyethylene-based system soaked in a bath with aqueous BA solution [35], where the catalytic effect is found to set in at approximately 1200 °C. This is fully in line with our results from Figure 5, where the catalytic effect is even visible from the very beginning of the carbonization at 1000 °C. Probably this low-temperature catalytic effect is achieved when boron-containing molecules, such as BA (or BA reaction products such as HBO_2_, H_2_B_4_O_7_ or B_2_O_3_), are chemically bound to the precursor (or its successor) at an early stage of conversion, thus increasing the interaction with the surrounding material when volatiles are ejected.

**Figure 6:**
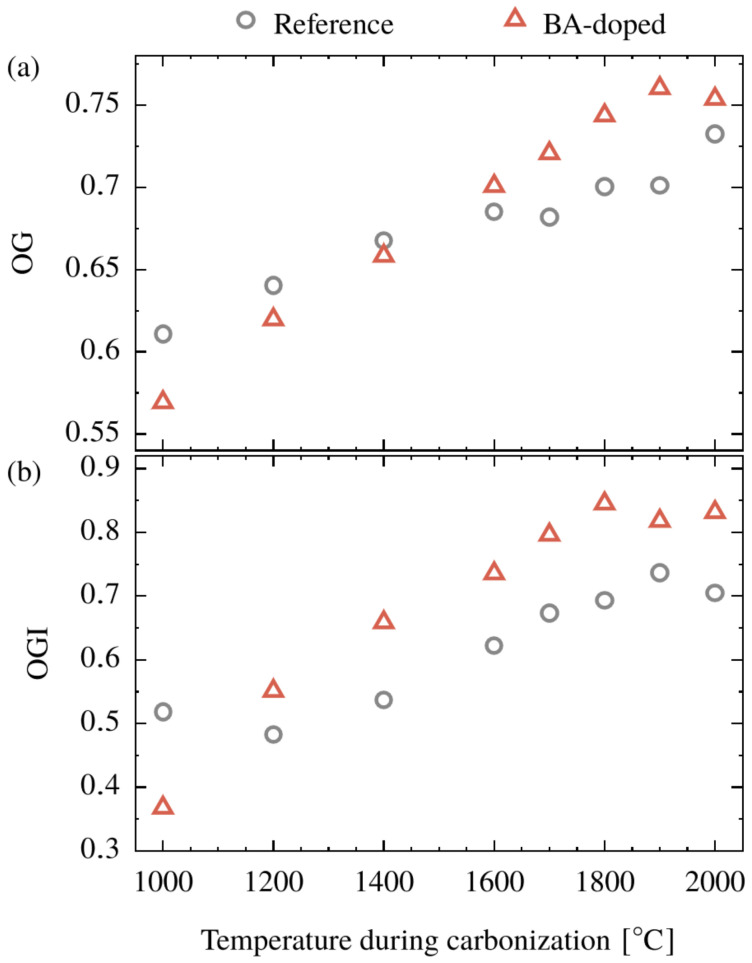
Degree of preferred relative orientation (*OG*) (**a**) and the integrated orientation parameter (*OGI*) (**b**) of a cellulose (Reference) and BA-doped precursor (Pre) as a function of the applied temperature during conversion.

In addition to the nature of the graphite-like crystallites, their orientation with respect to the fiber axis is also a relevant structural parameter governing the macroscopic properties of the resulting CFs, in particular the Young’s modulus. The orientation was determined from azimuthal scans of the (020) and (002) reflections for the precursors having cellulose II crystal structure and the CFs, respectively. In addition to the degree of preferred relative orientation (*OG*, Equation (5)), the integrated orientation parameter (*OGI*, Equation (6)) was calculated, and the results are shown in Table 1 and Figure 6. As to the precursors, no differences in *OG* = 0.80 and *OGI* = 0.20 were found; the additivation performed by an aqueous BA treatment does not change the preferred orientation (Table 1).

During the conversion, the *OG* increases for both systems (Figure 6a). The *OG* of the BA-doped system starts at a lower level and there is a crossover at 1600 °C and a further increase up to 1800 °C. At higher temperatures, there is a certain leveling off for the boron-doped system ending at approximately 0.76. In contrast, the *OG* of the reference system increases up to 2000 °C and reaches a lower maximum value of 0.73.

The overall increase in the *OGI* during the conversion, Figure 6b, suggests the formation of oriented structures in both systems. For the BA-doped system, the *OGI* remains constant at 0.83 between 1800 and 2000 °C, indicating that the transformation is completed. This final level is higher compared to the reference system, which reached an *OGI* of 0.72.

By comparing both systems, it can be concluded that for temperatures above 1600 °C, both *OG* and the *OGI* are increased when BA is used. This means (i) that the formation of an oriented graphite-like structure is promoted by boron (*OGI*) and (ii) that the orientation itself is increased (*OG*). Apart from the action of boron, the general increase in *OG* (and *OGI*) is remarkable since the carbonization was performed without stretching, i.e., no tension was applied.

### 3.4. Filament Cross Section

**Figure 7:**
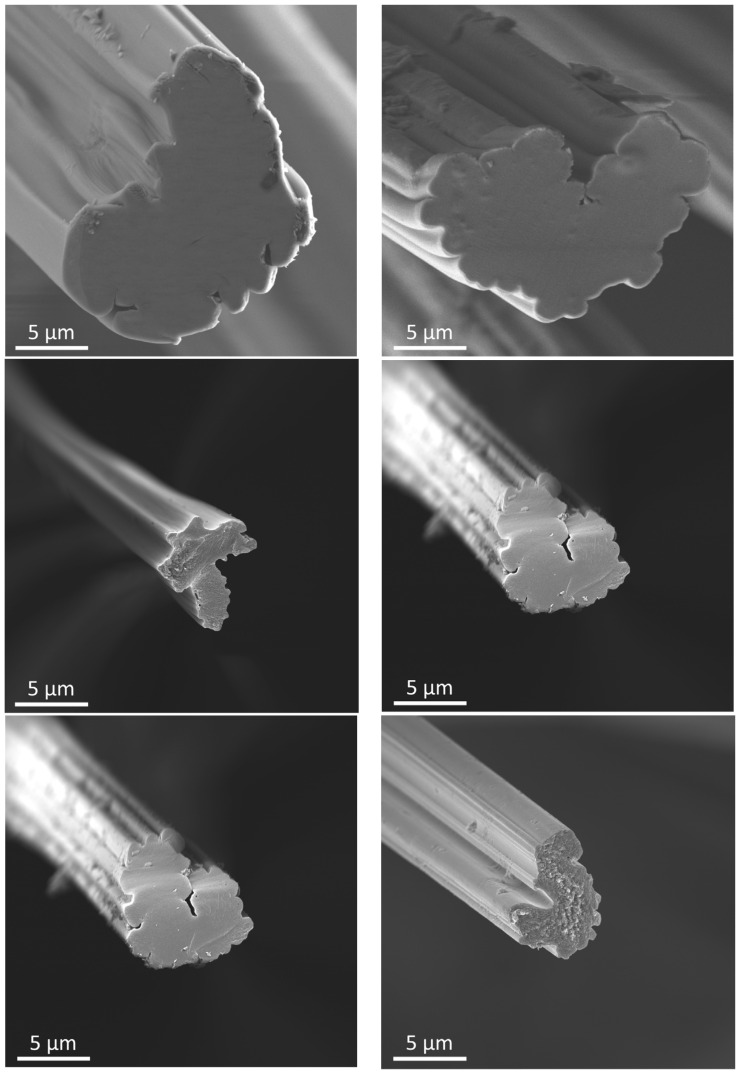
SEM Images of the single filament cross section: The left column shows the reference and the right column shows the BA-doped system. Precursor (**top**), CFs produced at 1000 °C (**middle**) and at 2000 °C (**bottom**) at the magnification of 3000×.

As shown in Figure 7, both precursors, the reference as well as BA-doped counterpart, are characterized by a strongly lobulated cross-sectional shape, which remains unchanged over the whole conversion process. It is clear that the fiber shape is set during the spinning process under the applied precipitation conditions and cannot be changed afterwards.

While the cross sections of both precursor systems show no noticeable voids, isolated macrospores (>50 nm) can be found after the carbonization at 1000 °C, which are preserved during carbonization at 2000 °C. These pores can only be found at the shell region of the cross sections and are most likely caused by the emission of volatile decomposition byproducts mainly formed below 1000 °C.

On the surfaces of the single filaments, deposits and particles can be found for all process stages. This effect is much more pronounced for the BA-doped system and can be assigned to BA deposits formed during the aqueous boric acid impregnation after spinning. Such deposits remain on the fiber surface over the whole conversion process and are converted later to B_4_C, which was proven by REM-EDX. 

**Figure 8:**
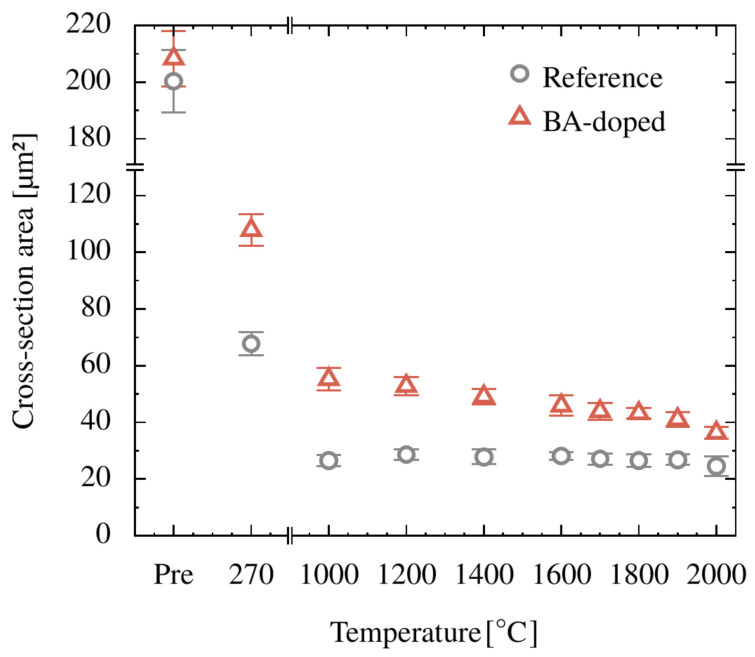
Average single filament section area of a cellulose (Reference) and BA-doped precursor (Pre) as a function of the applied temperature during conversion.

The detailed development of the cross-sectional area is shown in Figure 8. As expected, a remarkable decrease in the cross-sectional area with increasing temperature was found, which is in strong correlation with the residual mass. The impact of BA doping is clearly illustrated (Figure 7) by the SEM images for CFs produced at 1000 (middle row of Figure 7) or 2000 °C (bottom row). The higher residual mass for the BA-doped fibers results in a significantly larger cross-sectional area of 36 µm^2^ (d = 6.8 µm) compared to 27 µm^2^ (d = 5.8 µm) for the reference after carbonization at 2000 °C.

### 3.5. Density

**Figure 9:**
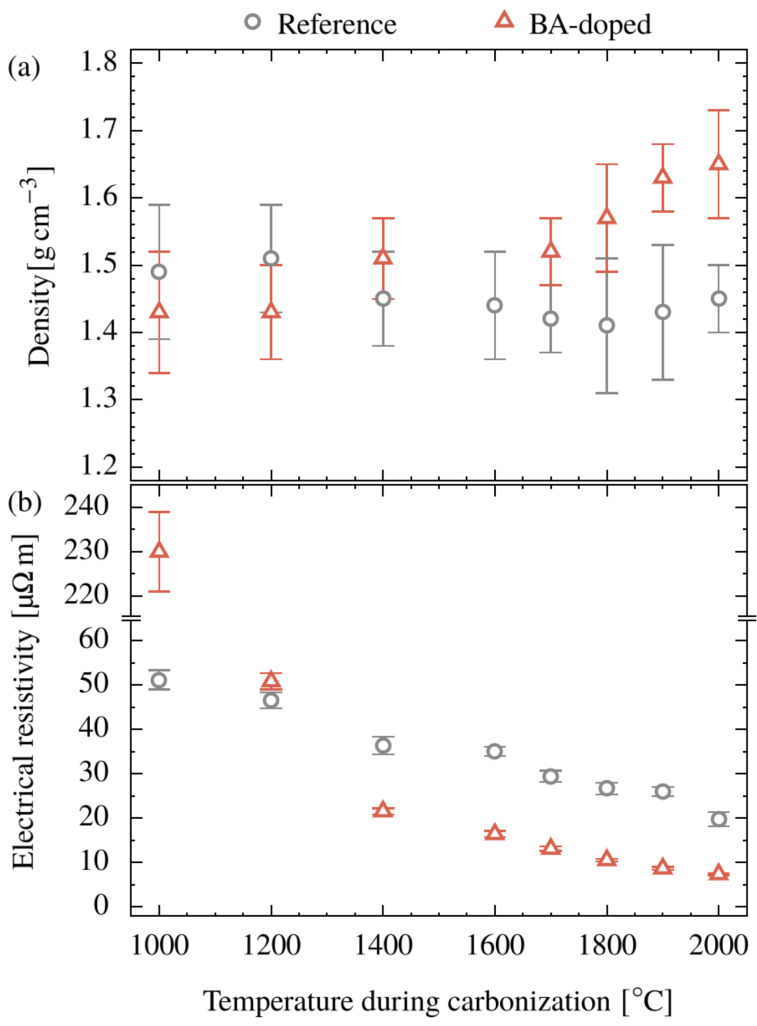
Density determined via density-gradient column (**a**) and electrical resistivity (**b**) of the CFs made from a cellulose-based (Reference) and BA-doped precursor as a function of the carbonization temperature.

Density results are shown in Figure 9a. The applied method (density-gradient column, DGC) only allows access of open pores by the column liquid such that the values given are affected not only by the density of the graphite-like phase but also by the volume fraction of the closed pores. Nevertheless, some conclusions can be drawn assuming the closed pores (if there are any) have a similar volume change, like the open ones. First, the density of the reference system remains rather constant at 1.45 g/cm^3^ over the temperature range investigated. Second, the density of the BA-doped system increases monotonously and crosses over the reference at 1700 °C. Third, between 1700 and 2000 °C, the density of the BA-doped system increases more rapidly and finally reaches a value of 1.65 g/cm^3^. So, the density follows the trend of crystallite growth, indicating that the progress of graphitization affects the density in a distinct manner.

In comparison with other cellulose-based CFs having a density between 1.35 and 1.5 g/cm^3^ [1,9], the reference system is within the expected range. The density for the BA-doped system of 1.65 g/cm^3^ is significantly higher.

### 3.6. Electrical Properties

In general, the electrical resistivity decreases during the carbonization for both systems (Figure 9b). There is a remarkable difference between the reference (50 µΩm) and doped system (230 µΩm) at 1000 °C. In the doped system, much more oxygen (15 mg/m) is present than in the reference (3 mg/m), Figure 3b. Since conductivity is driven by a certain connectivity of the carbon atoms, the oxygen may disrupt this connection. This would hold true also at 1200 °C, where the above oxygen values are 1.4 and 11 mg/m, but where the conductivities (resistivities) are equal. A second effect must come into play, probably the increase in *OGI* (Figure 6b), reflecting the amount of oriented material. With the complete loss of oxygen at 1400 °C, this effect becomes the only one and resistivities for the doped system are lower throughout. So, the overall slight decrease, starting from 1000 °C for the reference and from 1400 °C for the BA-doped system, for each, should be explained by the increase in crystallite sizes and crystallite orientation. 

The electrical resistivity of < 20 µΩm found for the reference CF carbonized at 2000 °C is remarkably low compared to the other cellulose-based CFs, ranging from 30 to 70 µΩm [9]. In terms of the BA-doped system, the electrical resistance drops further down to 7.4 µΩm representing an outstanding performance which is comparable to much more expensive pitch-based carbon fibers (e.g., DIALEAD K1352U: 6.6 µΩm [1]).

Along with electrical conductivity, thermal conductivity is another relevant macroscopic property of CFs. While the electronic contribution is decisive for conducting electricity, heat conduction has an additional contribution from phonons. From pitch-based CFs, it is known [1] that along with increasing electrical conductivity the phonon contribution increases as well. Thus, the conclusion may be drawn that, in the present case, the boron-doped CFs have higher thermal conductivity than the reference fibers.

### 3.7. Mechanical Properties

**Figure 10:**
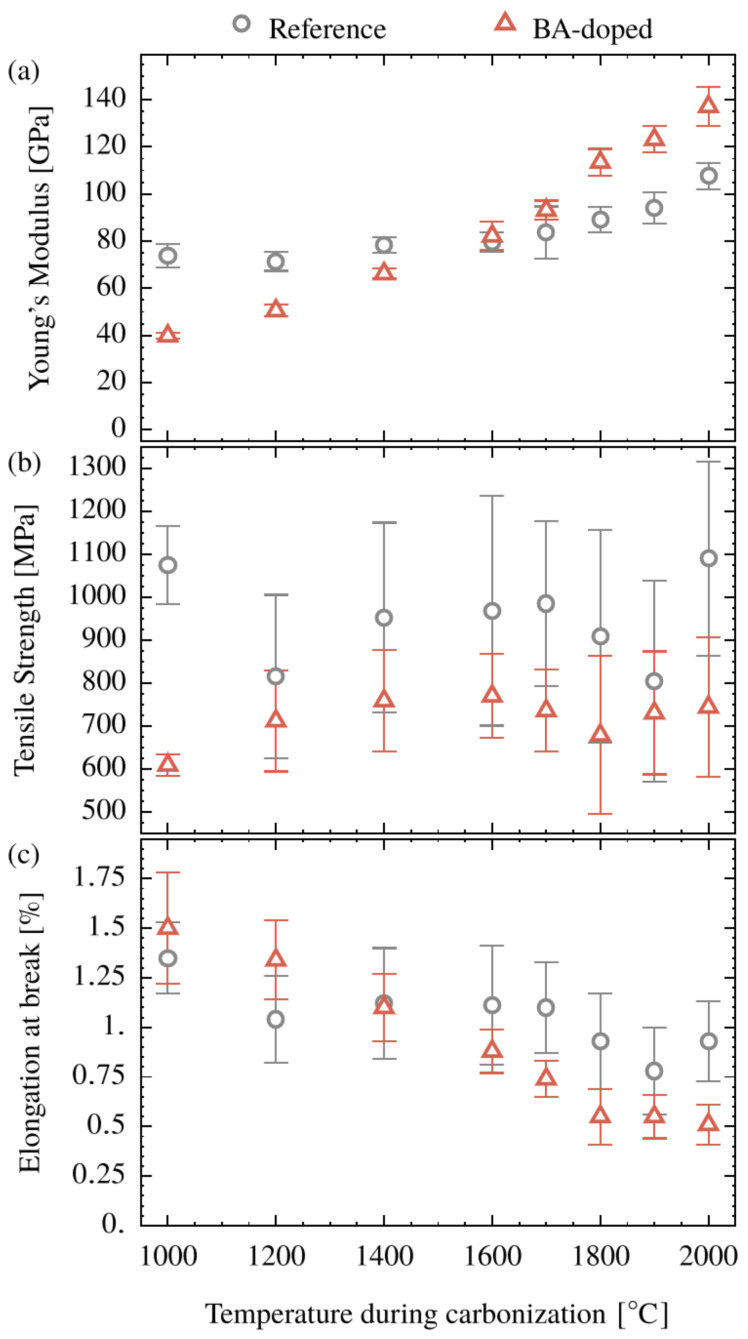
Young’s modulus (**a**), tensile strength (**b**) and elongation at break (**c**) of the CFs made from the cellulose (Reference) and the BA-doped precursor as a function of the applied carbonization temperature.

The stress-strain obtained by single filament tensile tests shows an almost linear behavior up to failure. The average values of tensile strength, Young’s modulus and elongation at break, are summarized in Figure 10. The Young’s modulus of the reference CFs increases in the temperature range from 1000 to 2000 °C from 73 to 108 GPa. The CFs based on the BA-doped system show a similar trend, but the starting level of 39 GPa at 1000 °C is significantly lower. Furthermore, the following temperature-induced increase is almost linear and much more pronounced. So, a crossover with the reference occurs at a temperature of 1600 °C and a maximum in the Young’s modulus of 137 GPa at 2000 °C is reached. 

**Figure 11:**
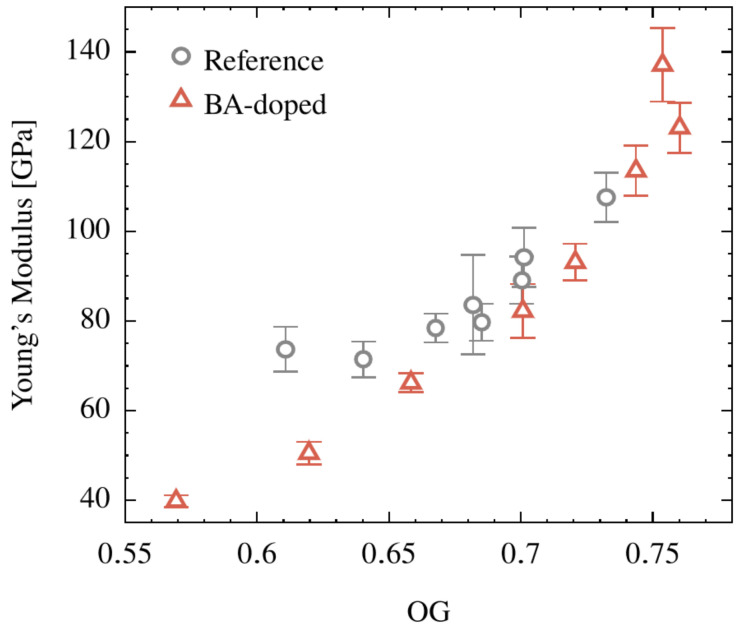
Young’s modulus as a function of the degree of preferred relative orientation *OG* for the CFs made from the cellulose (Reference) and the BA-doped precursor.

Correlating this macroscopic property, the Young’s modulus, with structural properties, reveals the following. Neither *d*_002_, nor *L_c_* or *L_a_* yields any obvious trend. In contrast, a correlation is detected between crystallite orientation and the Young’s modulus, as shown in Figure 11. This is in accord with the literature [1,35,56,57]. Many factors contribute to this tendency: the preferred orientation of the crystallites along the fiber axis, the growth of the oriented crystallites, and the decreasing heteroatom concentration.

In contrast to the Young’s modulus, tensile strength is generally not driven by preferred orientation, but is affected by structural features, such as voids, pores, and flaws in the bulk, as well as at the surface of the CFs. In the present case (Figure 10b), the values for tensile strength remain almost constant for each system over the entire carbonization temperature range. The level of the reference with 1000 MPa is significantly higher than that for the doped system, 750 MPa. This drop in strength must be explained by a particular structural issue. As seen in Figure 4c, crystals of an additional phase (presumably boron carbide) appear in the course of the carbonization indicating the presence of crystallizable domains, which disrupt the regular build-up of the carbonaceous structure, thus lowering the strength. SEM-EDX measurements at the fiber surface (not presented here) also indicate the presence of boron carbide. Another factor influencing strength is the tested volume (Weibull statistics). Since the clamping distance is identical, the higher cross section of the doped fibers induces a higher tested volume and thus a higher failure probability, if strength is defect driven as it is normally the case in carbon fibers.

From the fact that the stress–strain curves are straight lines, elongations at break provide no further independent information and are presented here for the sake of completeness in Figure 10c. 

In summary, BA doping results in an increase in the Young’s modulus from 108 to 137 GPa, with a simultaneous reduction in strength to about 750 MPa for the CFs carbonized at 2000 °C. Although the catalytic effect of boron on the structure formation starts already at 1000 °C, a noteworthy improvement in the Young’s modulus over the reference was observed only above 1600 °C.

## 4. Conclusions

A continuous process for the manufacturing of multifilament, cellulose-based carbon fibers (CFs) starting from spinning, followed by boric acid (BA) modification, stabilization at approximately 270 °C, low-temperature carbonization (1000 °C), and high-temperature carbonization (1000–2000 °C) was successfully developed. Chemical and physical structure formation, as well as the resulting mechanical and electrical properties, were studied as a function of the applied carbonization temperature both for the reference without BA treatment as well as the BA-doped system. It was shown that the inline impregnation with an aqueous boric acid solution in a treatment bath after spinning and before drying the precursor is an efficient way for the incorporation of a relevant BA fraction of approximately 6 wt% into the precursor. This BA treatment simultaneously allows for overcoming two major drawbacks of the cellulose-based CFs’ manufacturing, viz. the low carbon yield and the need for stretch graphitization. On one hand, the doping with BA leads to a remarkable increase in the carbon yield from 30 to 51%. On the other hand, the low-temperature catalytic effect of boron significantly accelerates the progress in graphitization during the carbonization process. The structural development of the crystalline phase was quantified by an increase in the crystallite dimensions bundle thickness *L_c_* and persistence length *L_a_* from 1.2 nm to 3.3 nm, respectively, for the reference of *L_c_* = 4.4 nm and *L_a_* = 8.8 nm, respectively, for the BA-doped system. The strong crystallite growth also affects the preferred relative orientation which increases gradually up to a degree of orientation *OG* = 0.76. Most relevant is the fact that the catalytic effect of boron already starts at 1000 °C. It becomes more and more effective with increased temperature reaching a maximum within the range of 1800 to 2000 °C. At the same time, the lattice plane spacing *d*_002_ is gradually decreased and finally reaches *d*_002_ = 0.3405 nm which is in the region of turbostratic carbon. 

Accompanying the progress in structural ordering, the macroscopic properties, such as Young’s modulus, electrical conductivity, and density, are increased. At 2000 °C, the Young’s modulus is improved by 27% from 108 to 137 GPa and electrical resistivity is decreased by 63% from 19.8 to 7.4 µΩm, compared to the reference system. On the downside, the strength is affected by 25% by BA doping, which is explained by the formation of additional structural defects, e.g., boron carbide.

To the authors’ knowledge, a Young’s modulus as high as 137 GPa is obtained in the tension-free processing of cellulose-based carbon fibers for the first time. However, as will be shown in a forthcoming paper, the BA-doped system offers the potential to achieve Young’s moduli up to 230 GPa by inducing a higher orientation of the crystallite phase.

## Figures and Tables

**Table 1:** Characteristics of the cellulose (Reference) and the BA-doped precursor used for the production of CFs.

Sample-ID	Reference	BA-Doped
spinning process	Viscose	Viscose
number of filaments	1000	1000
yarn linear density [tex]	294 ± 5	311 ± 5
single filament linear density [dtex]	2.9 ± 0.1	3.1 ± 0.1
single filament cross section [µm^2^]	200 ± 11	208 ± 10
single filament diameter ^1^ [µm]	16.0 ± 3.7	16.3 ± 3.6
boron content [wt%]	0.0 ± 0.1	1.0 ± 0.1
BA content ^2^ [wt%]	0.0 ± 0.1	5.6 ± 0.1
*OG* ^3^	0.80	0.79
*OGI* ^4^	0.20	0.20

^1^ calculated assuming round cross sections, ^2^ calculated from boron content, ^3^ degree of preferred relative orientation, ^4^ integrated orientation parameter.

## Data Availability

The data that support the findings of this study are available from the corresponding author, upon reasonable request.

## Supplementary Materials

The following supporting information can be downloaded at: https://www.mdpi.com/article/10.3390/polym15214310/s1, Figure S1: Experimental setup used for the stabilization and the low-temperature carbonization. Along the process chain: unwinder (1), stabilization furnace with three zones (2), tension meter for the stabilization (3), low-temperature furnace with 3 zones (4), tension meter for the low-temperature carbonization (5), rewinder (6), roller for fiber transport (a–c); Figure S2: Temperature profile during high-temperature carbonization; Figure S3: Experimental setup used for the high-temperature carbonization. Along the process chain: unwinder (1), high-temperature furnace with 6 zones (2), tension meter (3), rewinder (4), roller for fiber transport (a, b).
