# Supplementary material for: Boric Acid as A Low-Temperature Graphitization Aid and Its Impact on Structure and Properties of Cellulose-Based Carbon Fibers
Source: Polymers (Basel). 2023 Nov 2;15(21):4310. doi: 10.3390/polym15214310 (PMC10648134; doi:10.3390/polym15214310)
Supplement: Supplementary file 1 [file polymers-15-04310-s001.zip › polymers-2665454-supplementary.pdf]

Supplementary Information

Boric Acid as A Low-Temperature Graphitization Aid and Its Impact on Structure and Properties of Cellulose-Based Carbon Fibers

Tobias Hückstaedt *, Jens Erdmann, André Lehmann, Robert Protz and Johannes Ganster

Material Development and Structure Characterization, Biopolymers, Fraunhofer Institute for Applied Polymer Research IAP, Geiselbergstraße 69, 14476 Potsdam, Germany; jens.erdmann@iap.fraunhofer.de (J.E.); andre.lehmann@iap.fraunhofer.de (A.L.); robert.protz@iap.fraunhofer.de (R.P.); johannes.ganster@iap.fraunhofer.de (J.G.)

* Correspondence: tobias.hueckstaedt@iap.fraunhofer.de

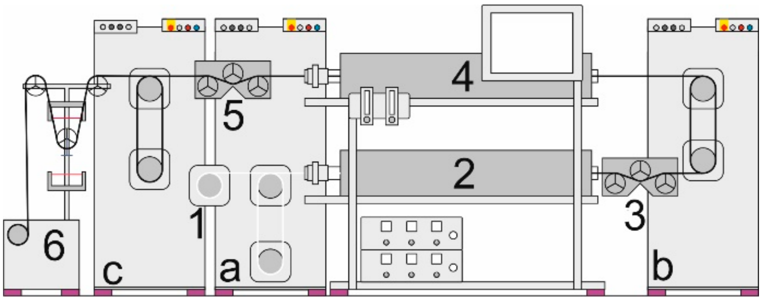

Figure S1. Experimental setup used for the stabilization and the low-temperature carbonization. Along the process chain: unwinder (1), stabilization furnace with three zones (2), tension meter for the stabilization (3), low-temperature furnace with 3 zones (4), tension meter for the low-temperature carbonization (5), rewinder (6), roller for fiber transport (a–c).

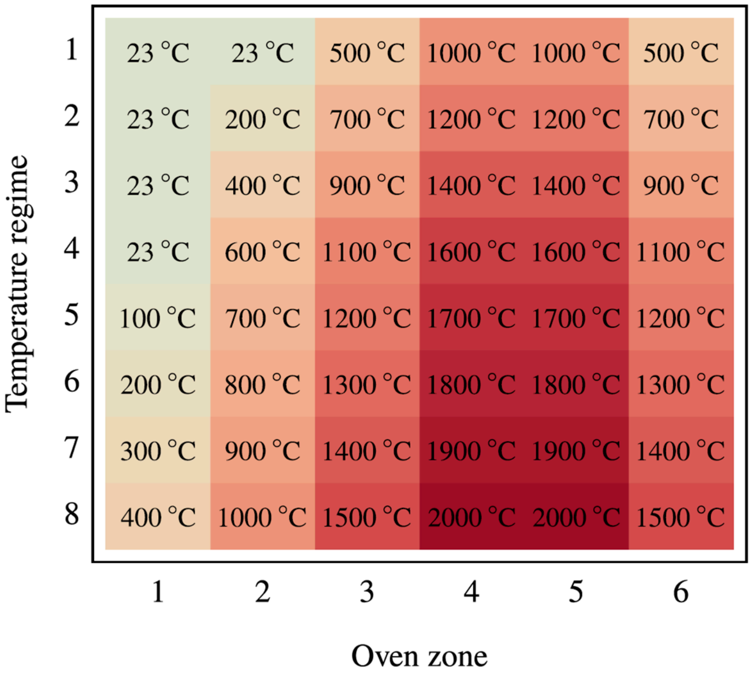

Figure S2. Temperature profile during high-temperature carbonization.

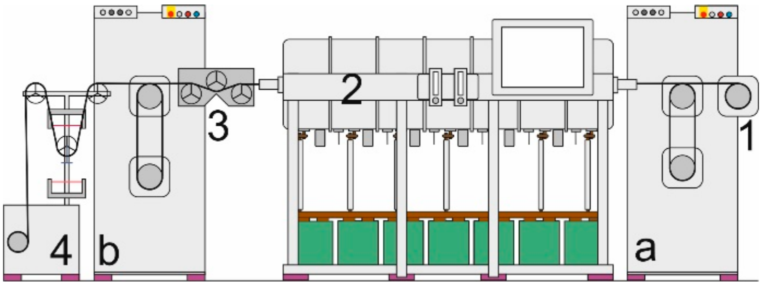

Figure S3. Experimental setup used for the high-temperature carbonization. Along the process chain: unwinder (1), high-temperature furnace with 6 zones (2), tension meter (3), rewinder (4), roller for fiber transport (a, b).
